# E17K substitution in AKT1 in prostate cancer

**DOI:** 10.1038/sj.bjc.6605673

**Published:** 2010-04-20

**Authors:** J L Boormans, H Korsten, A C J Ziel-van der Made, G J L H van Leenders, P C M S Verhagen, J Trapman

**Affiliations:** 1 Department of Urology, Erasmus University Medical Centre, PO Box 2040, 3000 CA, Rotterdam, The Netherlands; 2 Department of Pathology, Erasmus University Medical Centre, Josephine Nefkens Institute, PO Box 2040, 3000 CA, Rotterdam, The Netherlands

**Keywords:** AKT1, E17K, mutation, PI3K, prostate cancer

## Abstract

**Background:**

The phosphatidylinositol 3-kinase (PI3K)–AKT pathway is activated in many cancers. Mutational hotspots in *AKT1* and in the regulatory and catalytic subunits of *PI3K* have been detected in multiple tumour types. In AKT1, the E17K substitution leads to a PI3K-independent activation of AKT1.

**Methods:**

A mutational profiling of *AKT1* and of the mutational hotspots in *PIK3CA* and *PIK3R1* was carried out in samples from primary and recurrent prostate tumours.

**Results:**

We show that, in prostate cancer, AKT1(E17K) had a prevalence of 1.4%. The mutation seemed to be associated with a favourable clinical course but it was not associated with a specific tumour growth pattern. Activating mutations in *PIK3CA* or *PIK3R1* were not found in prostate cancer.

**Conclusion:**

The E17K substitution in AKT1 is rare in prostate cancer. It seems associated with a favourable clinical outcome but not with a specific histology of the tumour.

The phosphatidylinositol 3-kinase (PI3K)–v-akt murine thymoma viral oncogene homologue (AKT) signalling pathway is involved in cellular processes such as cell growth, proliferation, apoptosis, and cytoskeletal rearrangement. PI3K functions by catalysing the production of phosphorylated phosphoinositides (PtdIns). The PI3K phosphorylates PtdIns(4,5)P2 (PIP2) into PtdIns(3,4,5)P3 (PIP3), which binds to the pleckstrin homology domain of the downstream target, v-akt murine thymoma viral oncogene homologue 1 (AKT1). This results in a recruitment of AKT1 to the plasma membrane in which regulatory amino-acid residues serine 473 (Ser473) and threonine 308 (Thr308) are phosphorylated and activated (Vivanco and Sawyers, 2002).

The tumour-suppressor gene phosphatase and tensin homologue (*PTEN*) directly antagonises the PI3K–AKT pathway by converting PIP3 back to PIP2. Absence of PTEN leads to increased phosphorylation of AKT (Cantley and Neel, 1999), thereby stimulating PI3K–AKT signalling. *PTEN* inactivation is frequent in various cancers, including prostate cancer (Li *et al*, 1997). It can occur by deletion (Cairns *et al*, 1997; Vlietstra *et al*, 1998; Verhagen *et al*, 2006), mutation (Suzuki *et al*, 1998; Vlietstra *et al*, 1998; Verhagen *et al*, 2006), or by decreased expression (Whang *et al*, 1998). Prostate-targeted *Pten* knockout mice develop prostate hyperplasia, intraepithelial neoplasia, and ultimately invasive cancer (Wang *et al*, 2003; Ma *et al*, 2005).

Activating mutations in *AKT1* or *PI3K* are other mechanisms that lead to stimulation of the PI3K–AKT pathway. PI3K is a heterodimer composed of a regulatory subunit (p85*α*), encoded by *PIK3R1*, and a catalytic subunit (p110 *α*), encoded by *PIK3CA*. *PIK3CA* has frequently been reported as being mutated in various human cancers (Samuels *et al*, 2004; Levine *et al*, 2005). *PIK3R1* mutations are less common, although recently it was shown that *PIK3R1* was mutated in up to 10% of glioblastomas (Cancer Genome Atlas Research Network, 2008; Parsons *et al*, 2008). Mutations in either gene lead to a disruption of the interaction between the regulatory and catalytic subunits, which enhances enzymatic activity (Huang *et al*, 2007), thereby activating the PI3K–AKT pathway. In prostate cancer, however, activating mutations in *PIK3CA* or *PIK3R1* have not been reported thus far (Majumder and Sellers, 2005; Ligresti *et al*, 2009).

Recently, a unique mutation in the pleckstrin homology domain of *AKT1* was identified in breast, ovarian, and colorectal cancer (Carpten *et al*, 2007). This G>A mutation results in a lysine substitution for glutamate at position 17 (E17K) and leads to a PI3K-independent activation of AKT1. The mutation was mutually exclusive with respect to mutations in *PI3K* and loss of PTEN protein expression. Others confirmed the mutation in breast and colorectal tumours (Bleeker *et al*, 2008) and incidental cases were identified in bladder, endometrial, lung, and skin cancer (Davies *et al*, 2008; Do *et al*, 2008; Malanga *et al*, 2008; Zilberman *et al*, 2009; Shoji *et al*, 2009). An analogous E17K substitution in AKT3 has also been found in melanomas (Davies *et al*, 2008). Previously, we reported on the E17K substitution in AKT1 in a ductal adenocarcinoma of the prostate in a patient who had a very long cancer-specific survival (Boormans *et al*, 2008).

In this study, we analysed the prevalence of AKT1(E17K) in a larger cohort of prostate cancer patients. We investigated whether the E17K substitution in AKT1 was associated with a specific growth pattern of prostate cancer and whether it corresponded with clinical outcome.

## Materials and methods

### *AKT1*

Genomic DNA was available from 184 freshly frozen clinical prostate cancer samples. A total of 85 samples were primary prostate tumours obtained by radical prostatectomy, 88 samples were locally advanced or recurrent tumours obtained by transurethral resection of the prostate, and 11 samples were hormone-naïve prostate cancer lymph node metastases obtained by pelvic lymph node dissection. An additional 30 formalin-fixed, paraffin-embedded primary prostate tumours were selected, including 18 ductal adenocarcinomas of the prostate and 12 primary prostate tumours with the following characteristics: pathological T stage ⩾ pT3a, prostate-specific antigen level ⩾4.0 ng *μ*l^−1^, any Gleason score, and a cancer-specific survival of >13 years. From the cancerous regions of the paraffin-embedded samples, 1-mm core biopsy samples were taken (Beecher Instruments, Silver Spring, MD, USA). In mutated samples, a core biopsy sample was taken from adjacent benign prostatic tissue to test whether the mutation was truly somatic. Genomic DNA was isolated using the Puregene DNA isolation kit (BIOzym, Landgraaf, the Netherlands), according to the manufacturer's instructions.

PCR analysis was carried out to yield a 198-bp genomic fragment of *AKT1* (see Supplementary Table 1 for primer sequences). PCR products were identified by 2% agarose gel electrophoresis and ethidium bromide staining. After ExoSapI (USB, Staufen, Germany) treatment, the PCR fragments were sequenced with the reverse *AKT1* primer. Sequence reaction products were analysed on the ABI 3700 automated DNA sequencer (Applied Biosystems, Carslbad, CA, USA). The freshly frozen samples containing the E17K substitution in AKT1 were also analysed for *PTEN* mutations and for mutations in the mutational hotspots of exons 9 and 20 of *PIK3CA* (see Supplementary Table 1 for primer sequences of *PIK3CA* and *PTEN*).

### *PIK3CA* and *PIK3R1*

Mutational analysis of *PIK3CA* and *PIK3R1* was also carried out on a subset of 63 freshly frozen tissue samples: 61 transurethral resection of the prostate samples and two lymph node metastases. The exons known to contain mutational hotspots were sequenced, that is, exons 9 and 20 for *PIK3CA* and exons 14 and 15 for *PIK3R1* (see Supplementary Table 1 for primer sequences).

## Results and discussion

The PI3K–AKT signalling pathway is a central factor in various cellular processes that are involved in carcinogenesis. The E17K substitution in AKT1 is an important mechanism that leads to a PI3K-independent activation of AKT1 (Carpten *et al*, 2007). Rare E17K substitution in AKT3 has also been described (Davies *et al*, 2008). In this study, we focussed on the more common AKT1(E17K) substitution in prostate cancer. Previously, we identified AKT1(E17K) in a pure ductal adenocarcinoma of the prostate in a patient who had a long survival (Boormans *et al*, 2008). Here, we analysed an additional 18 ductal prostate cancer samples; however, none of these samples contained the E17K substitution in AKT1. Therefore, we concluded that an association between AKT1(E17K) and a specific ductal growth pattern of prostate cancer is unlikely. This is in contrast to AKT1(E17K) in breast and lung cancer. In breast cancer, the mutation was unique for lobular and ductal histotypes (Bleeker *et al*, 2008), whereas in lung tumours AKT1(E17K) was only seen in squamous cell carcinomas (Bleeker *et al*, 2008; Do *et al*, 2008; Malanga *et al*, 2008).

Next, we randomly extended our search for AKT1(E17K) in 184 freshly frozen clinical prostate cancer samples. One extra patient harbouring the mutation was identified. The sample was a primary prostate tumour obtained by radical prostatectomy. The tumour was a moderately differentiated adenocarcinoma (Gleason score 3+3=6) with bladder neck involvement (pathological T stage: pT4a) and positive surgical margins. Moreover, occult pelvic lymph node metastases were present at the time of radical prostatectomy. Despite these prognostic unfavourable characteristics, the patient is still alive at the end of follow-up (survival ∼17 years). These findings were in agreement with the clinical course of the patient we described in our previous report (Boormans *et al*, 2008). That patient was diagnosed with a poorly differentiated adenocarcinoma (Gleason score 4+4=8), but he also had a long survival (>18 years) and he did not die from prostate cancer (see Table 1a for the clinical and histopathological characteristics of the patients).

To investigate whether AKT1(E17K) in prostate cancer was associated with a favourable clinical outcome, as suggested by the first two patients harbouring the *AKT1* mutation, we selected an additional 12 paraffin-embedded primary prostate tumours. All patients had unfavourable clinicopathological characteristics; nevertheless, they had a survival of >13 years (see Materials and Methods section for selection criteria). In these additional 12 prostate tumours, we identified one extra patient having the E17K substitution in AKT1. Pathology showed a moderately differentiated adenocarcinoma (Gleason score 3+3=6) with extracapsular extension (pathological T stage: pT3a) and positive surgical margins. Almost 18 years after the radical prostatectomy, the patient died from causes other than prostate cancer.

In a recent series from our institution on patients with clinical T3 prostate tumours who were treated by radical prostatectomy and pelvic lymphadenectomy, cancer-specific and overall survival after 15 years was 66 and 37%, respectively (Hsu *et al*, 2009). In a recent review, it was hypothesised that the presence of AKT1(E17K) is associated with a less-aggressive form of cancer (Brugge *et al*, 2007). The findings of our present series could be in agreement with such a hypothesis: all three patients harbouring the E17K substitution in AKT1 had a very long survival despite aggressive clinicopathological characteristics. Obviously, the findings of this study do not prove an association with better outcome because of the low prevalence of AKT1(E17K) in prostate cancer.

The mutational profiling of the mutational hotspots of *PIK3CA* and *PIK3R1* in a subset of 63 prostate cancer patients did not reveal any mutated sample, which is consistent with earlier findings (Ligresti *et al*, 2009).

In conclusion, we have identified three patients harbouring E17K substitution in AKT1 in prostate cancer, rendering the reported frequency for AKT1(E17K) in prostate cancer at 1.4% (3 out of 214 patients examined). The mutation showed to be truly somatic, as the sequence of *AKT1* derived from benign prostatic tissue of patients carrying the mutation was of wild type (data not shown). AKT1(E17K) seemed to be associated with a favourable clinical course, but no correlation with a ductal growth pattern of prostate cancer was found. Furthermore, the E17K substitution in AKT1 was mutually exclusive with respect to mutations in *PTEN* and *PIK3CA*, as in the freshly frozen samples, mutational profiling of all nine *PTEN* exons and of the mutational hotspots of *PIK3CA* (exons 9 and 20) showed no mutations (Table 1b). The possibility of loss of PTEN function by loss of expression was not assessed in this study.

## Figures and Tables

**Table 1a:** Clinical and histopathological characteristics of three prostate cancer patients harbouring the E17K substitution in AKT1

**Patient**	**Age at diagnosis (years)**	**Initial PSA (ng ml^−1^)**	**Primary treatment**	**Secondary treatment**	**Tertiary treatment**	**cT-stage**	**pT-stage**	**Gleason score**
I	74	8.8	WaWa	TURP	TURP+ET	cT2b	NA	4+4=8
II	72	13.0	RP and PLND	None	None	cT2b	pT4a	3+3=6
III	62	5.6	RP and PLND	None	None	cT3x	pT3a	3+3=6

**Patient**	**Surgical margins**	**N+**	**Tissue**	**Histotype**	**Death**	**OS (years)**	**PCa death**	**CSS (years)**
I	NA	NA	TURP freshly frozen	Ductal adenocarcinoma	Yes	18.4	No	⩾18.4
II	Positive	Yes	RP freshly frozen	Acinar adenocarcinoma	No	⩾17.0	No	⩾17
III	Positive	No	RP PEFF	Acinar Adenocarcinoma	Yes	16.8	No	⩾16.8

Abbreviations: CSS=cancer-specific survival; cT-stage=clinical T stage; ET=endocrine therapy; NA=not applicable; OS=overall survival; PCa death=prostate cancer death; PLND=pelvic lymph node dissection; PEFF=paraffin-embedded, formalin fixed; PSA=prostate-specific antigen; pT-stage=pathological T stage; RP=radical prostatectomy; TURP=transurethral resection of the prostate; WaWa=watchful waiting.

**Table 1b:** Genetic characteristics of three prostate cancer patients harbouring the E17K substitution in AKT1

**Patient**	**Sequence of *PTEN* exons**	**Sequence of *PIK3CA* exons 9 and 20**	**Sequence of *PIK3R1* exons 14 and 15**
I	Wild type	Wild type	Wild type
II	Wild type	Wild type	Unknown
III	Unknown	Unknown	Unknown

Abbreviation: *PTEN*=phosphatase and tensin homologue.
