# Supplementary material for: E17K substitution in AKT1 in prostate cancer
Source: Br J Cancer. 2010 Apr 20;102(10):1491–4. doi: 10.1038/sj.bjc.6605673 (PMC2869172; doi:10.1038/sj.bjc.6605673)
Supplement: Supplementary Table 1 [file 6605673x1.doc]

**Supplementary data**

**Supplementary Table 1. Primer sequences of primers used for PCR and sequence analysis**

| **Target** | **Forward 5'->3'** | **Reverse 5'->3'** |
| --- | --- | --- |
| *AKT1* | *tagagtgtgcgtggctctca* | *ctgaatcccgagaggccaa* |
| *AKT1 Seq* |  | *ctgaatcccgagaggccaa* |
| *PI3KCA exon 9* | *gattggttctttcctgtctctg* | *ccacaaatatcaatttacaaccattg* |
| *PI3KCA exon 9 Seq* | *ttgctttttctgtaaatcatctgtg* |  |
| *PI3KCA exon 20* | *tggggtaaagggaatcaaaag* | *cctatgcaatcggtctttgc* |
| *PI3KCA exon 20 Seq* | *tgacatttgagcaaagacctg* |  |
| *PI3KR1 exon 14 and 15* | *acttgaagaagcaggcagct* | *cattgcccaaccactcgtt* |
| *PI3KR1 exon 14 and 15 Seq* |  | *cattgcccaaccactcgtt* |
| *PTEN exon 1* | *agtcgctgcaaccatcc* | *ctaagagagtgacagaaaggta* |
| *PTEN exon 1 Seq* | *agtcgctgcaaccatcc* |  |
| *PTEN exon 2* | *ttagtttgattgctgcatatttc* | *acatcaatatttgaaatagaaaatca* |
| *PTEN exon 2 Seq* | *ttagtttgattgctgcatatttc* |  |
| *PTEN exon 3* | *tgttaatggtggctttttg* | *gcaagcatacaaataagaaaac* |
| *PTEN exon 3 Seq* | *tgttaatggtggctttttg* |  |
| *PTEN exon 4* | *ttcctaagtgcaaaagataac* | *tacagtctatcgggttaagt* |
| *PTEN exon 4 Seq* | *ttcctaagtgcaaaagataac* |  |
| *PTEN exon 5* | *gcaacatttctaaagttaccta* | *ctgttttccaataaattctca* |
| *PTEN exon 5 Seq* |  | *ctgttttccaataaattctca* |
| *PTEN exon 6* | *gaaataactataatggaaca* | *atggaaggatgagaatttcaagc* |
| *PTEN exon 6 Seq* | *gaaataactataatggaaca* |  |
| *PTEN exon 7* | *atcgtttttgacagtttg* | *tcccaatgaaagtaaagtaca* |
| *PTEN exon 7 Seq* | *atcgtttttgacagtttg* |  |
| *PTEN exon 8* | *tgcaaatgtttaacataggtga* | *cagctgtactcctagaatta* |
| *PTEN exon 8 Seq* |  | *cagctgtactcctagaatta* |
| *PTEN exon 9* | *gttcatctgcaaaatgga* | *ggtaatctgacacaatgtccta* |
| *PTEN exon 9 Seq* |  | *ggtaatctgacacaatgtccta* |

Abbreviations: Seq = primer used for sequence analysis
